# Supplementary material for: The interactome of intact mitochondria by cross-linking mass spectrometry provides evidence for coexisting respiratory supercomplexes
Source: Mol Cell Proteomics. 2017 Dec 8;17(2):216–32. doi: 10.1074/mcp.RA117.000470 (PMC5795388; doi:10.1074/mcp.RA117.000470)
Supplement: Supplemental Data [file supp_RA117.000470_133922_0_supp_23978_fzffwf.zip › spectra_annotation/mito_DR_spectra_annotation/402-1-15-1-19-1.pdf]

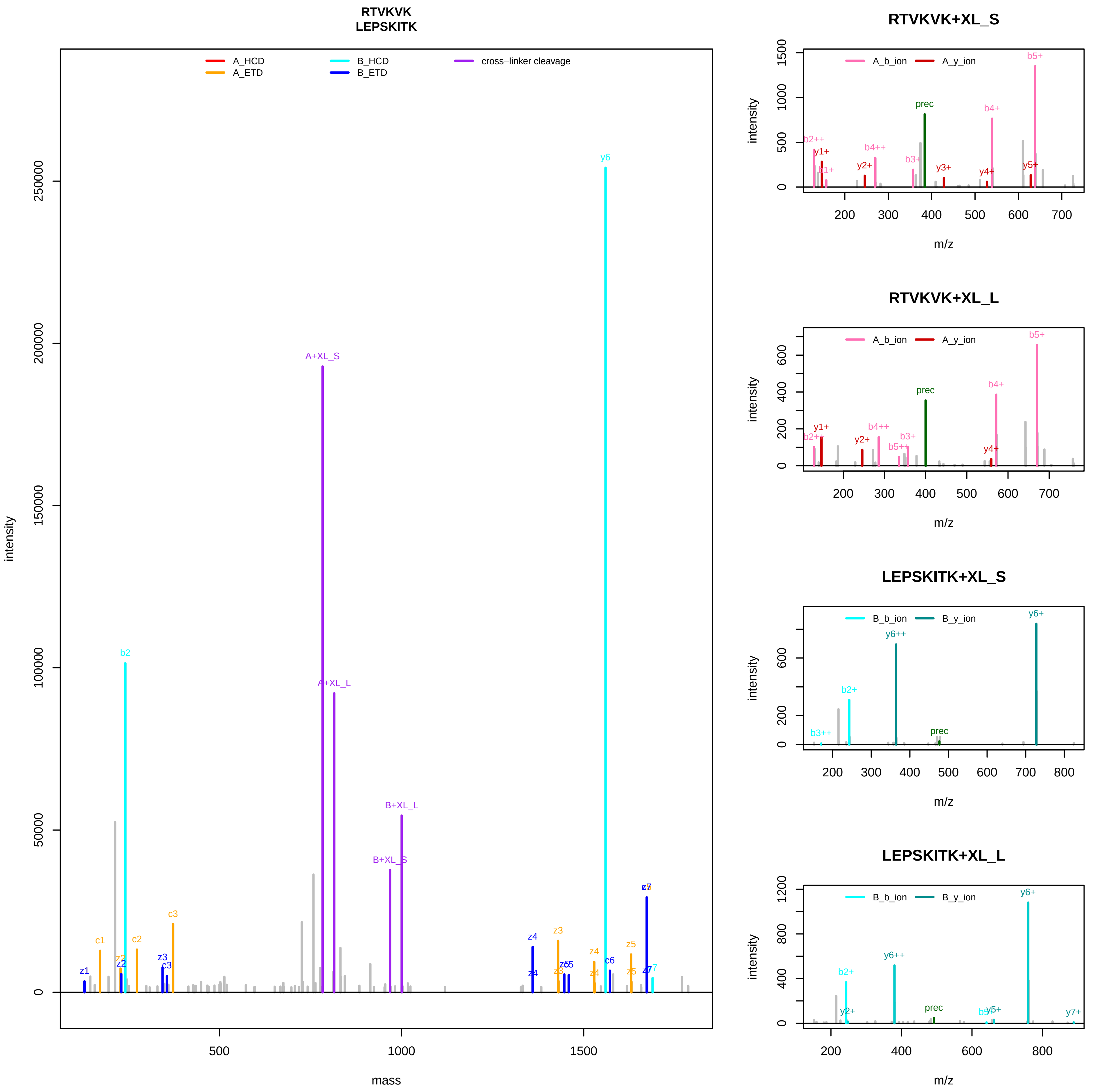
RTVKVK
LEPSKITK
A_HCD
A_ETD
B_HCD
B_ETD
cross−linker cleavage
intensity
mass
y6
A+XL_S
A+XL_L
B+XL_L
B+XL_S
b2
c1
c2
c3
z1
z2
z3
c3
z4
z3
z4
c6
z5
c7
z7
RTVKVK+XL_S
A_b_ion
A_y_ion
prec
b5+
b4+
b4++
b3+
b2++
y1+
b1+
y2+
y3+
y4+
y5+
m/z
RTVKVK+XL_L
A_b_ion
A_y_ion
prec
b5+
b4+
b4++
b3+
b5++
b2++
y1+
y2+
y4+
m/z
LEPSKITK+XL_S
B_b_ion
B_y_ion
y6+
y6++
b2+
b3++
prec
m/z
LEPSKITK+XL_L
B_b_ion
B_y_ion
y6+
y6++
b2+
y2+
prec
y5+
y7+
m/z
